# Modulation of influenza vaccine immune responses using an epidermal growth factor receptor kinase inhibitor

**DOI:** 10.1038/srep12321

**Published:** 2015-07-31

**Authors:** Joanna A. Pulit-Penaloza, Bishu Sapkota, E. Stein Esser, Richard W. Compans, Brian P. Pollack, Ioanna Skountzou

**Affiliations:** 1 Department of Microbiology and Immunology and Emory Vaccine Center, Atlanta, GA, 30322; 2 Atlanta Veterans Affairs Medical Center, Decatur, GA 30033; 3 Department of Dermatology, Emory University, Atlanta, GA 30322; 4 Winship Cancer Institute, Emory University, Atlanta, GA 30322.

## Abstract

Systemic use of epidermal growth factor receptor inhibitors (EGFRIs) has been shown to alter MHC expression and that of several chemokines, and to enhance immune cell recruitment into human skin. We hypothesized that EGFRIs may have value as cutaneous immune response modifiers, and determined the effects of topical application of an irreversible EGFRI on a well-established murine model of influenza vaccination. We found that a single topical application of an EGFRI led to increased levels of antibodies that inhibit influenza mediated hemagglutination and viral cytopathic effects. The topically applied EGFRI significantly enhanced the generation of vaccine-specific IL-4 and IFN-γ producing cells within skin-draining lymph nodes as early as one week following vaccination. The EGFRI/vaccine group showed a twelve-fold reduction in detectable pulmonary viral load four days after infection as compared to the vaccine alone control group. The reduction in the lung viral titers correlated with the survival rate, which demonstrated 100% protection in the EGFRI/vaccine immunized group but only 65% protection in the mice immunized with vaccine alone. These findings are significant because they demonstrate that inhibition of defined signaling pathways within the skin using small molecule kinase inhibitors provides a novel approach to enhance immune responses to vaccines.

Vaccines are one of the most cost-effective medical interventions, with a profound benefit to mankind. Since their widespread introduction in the 20^th^ century they are estimated to have prevented almost 6 million deaths per year, saved 386 million life years and saved more than 20 million children’s lives over the past 20 years^1^,^2^. Safety and tolerability concerns accompanying the use of vaccines that incorporate live-attenuated or killed microorganisms have led to the increasing use of vaccines composed of pathogen subunits. A drawback of this approach is that such subunit-based vaccines lack some of the inherent immunostimulatory properties of whole organism-based vaccines^3^. As such, they result in suboptimal humoral responses and low or no T cell responses, making multiple immunizations necessary to induce protective immunity^3^. Identification of effective adjuvants and alternative routes of immunization are important to overcome these challenges. Such advances have the potential to reduce the burden of re-vaccination and enhance vaccine efficacy, particularly in young, aged and immunocompromised populations.

Currently used adjuvants consist of compounds that are co-injected with vaccine antigens, and include a variety of aluminum salts, immunostimulatory molecules, and emulsions containing oil and water^4^. While these adjuvant approaches can enhance the immune response to some vaccines, they are not universally effective. Moreover, because they are rarely studied in the absence of antigen, there are limited studies to define their mechanistic underpinnings^5^. In addition, the complexity and large molecular size of many adjuvants may hinder their incorporation into less invasive vaccine delivery strategies.

The delivery of vaccine components through the skin via intradermal, subcutaneous, and intramuscular injection are the most common routes of immunization. Despite the well characterized importance of the epidermis in regulating cutaneous immune responses, its relevance to vaccination has received little attention because it is largely bypassed during injection-based vaccinations. It is likely that as less invasive approaches of vaccination, such as those utilizing microneedles or nanoparticles become more widespread, the need to fully understand the role of the epidermis in the context of vaccination will gain importance^6^,^7^,^8^. Likewise the identification and incorporation of topical agents that can act on cellular elements of the epidermis is an important approach to augment vaccine responses to cutaneous immunization^9^.

The pressing need to develop approaches to enhance the response to influenza vaccination is widely recognized^10^. In addition, successful vaccination approaches using epicutaneous and microneedle delivery platforms have been thoroughly studied in models of influenza and there is evidence that perturbations of the epidermis can functionally influence the response to influenza vaccination^11^,^12^. In this study, we sought to determine if pharmacologic inhibition of signal transduction pathways could influence the response to influenza vaccination by coupling the local application of an epidermal growth factor receptor (EGFR) inhibitor (EGFRI) with intradermal administration of influenza vaccine.

We selected an inhibitor of the EGFR for the following reasons. Prior studies have shown that EGFRI therapy is associated with increased recruitment of immune cells such as dendritic cells and macrophages into the skin^13^. For our studies we selected an irreversible EGFRI (known as PD168393) because previous reports using murine models have shown that local application of this inhibitor to the skin enhances an antigen-specific cell-mediated immune response as well as blocks the immunosuppressive effects of ultraviolet radiation^14^,^15^. Consistent with their ability to influence cutaneous immune homeostasis, clinical trials using related irreversible EGFRIs such as dacomitinib and afatinib given systemically for the treatment of advanced cancer are associated with increased skin inflammation^16^,^17^. Thus, in both murine models and in clinical trials EGFRIs have been reported to influence immune cell trafficking and cutaneous immune homeostasis both of which are likely relevant to vaccination responses. Here, we demonstrate that topical application of a small molecule EGFR kinase inhibitor can be used to enhance the immune response to an intradermal influenza subunit vaccine. These data provide proof-of-concept evidence that kinase inhibitors delivered locally to the skin offer a novel approach to modulate cutaneous vaccine responses.

## Results

### Enhancement of humoral responses to influenza vaccine by the EGFRI PD168393

**Figure 1:**
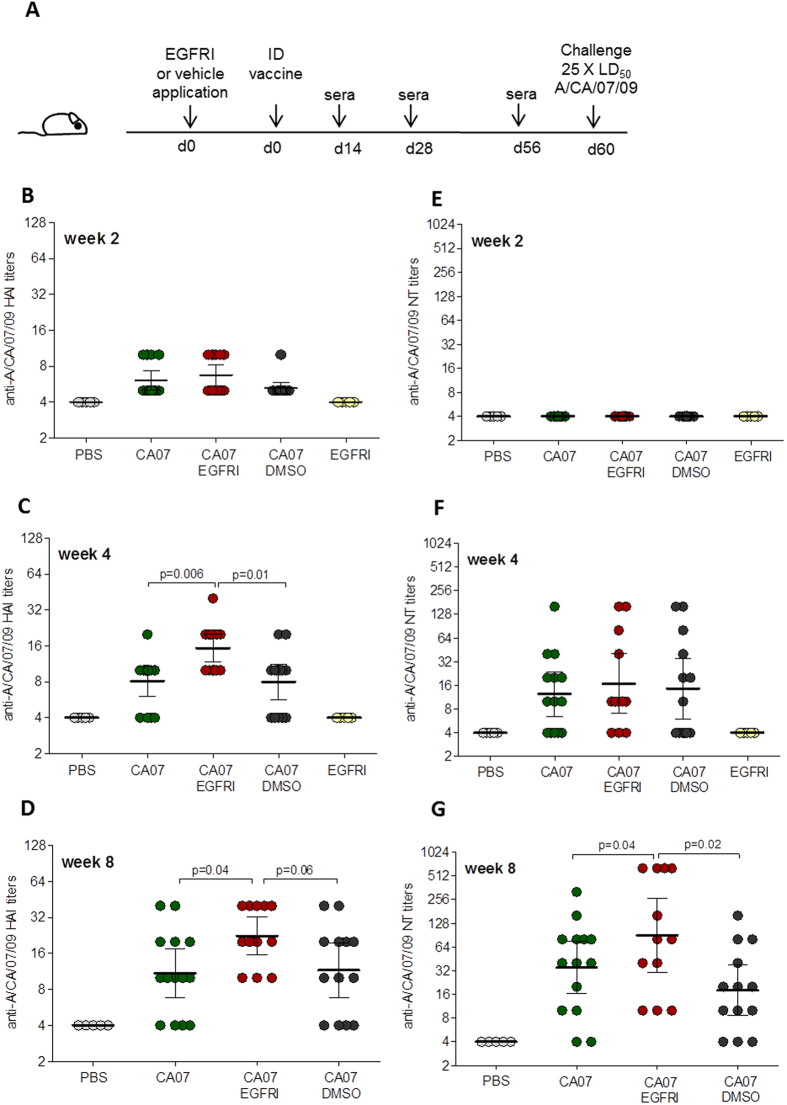
Humoral immune responses in mice topically pretreated with PD168393 prior to ID vaccination. (**A**) Six- to eight-week-old female BALB/c mice (mouse image drawn by Joanna Pulit-Penaloza) were shaved and then topically treated with 40 μl of 4 mM EGFRI (PD168393) or vehicle control (10% DMSO in ethanol) for 10 min prior to intradermal injection of A/California/07/09 subunit vaccine in the same area. Untreated mice intradermally vaccinated with A/California/07/09 were used as a positive control; unvaccinated mice injected with PBS or topically treated with PD168393 were used as negative controls. Animals were bled at weeks 2, 4 and 8 after vaccination and challenged with 25xLD_50_ of homologous virus 60 days after vaccination. Sera collected 2, 4 and 8 weeks post-vaccination were analyzed for (**B**–**D**) influenza specific hemagglutinin inhibition titers (HAI) and (**E**–**G**) neutralizing antibody titers (NT). Values are expressed as geometric mean with a ±95% confidence interval (n = 13–14). CA07; A/California/07/09.

To determine the impact of a topical EGFRI on the response to influenza vaccination, we applied PD168393 prior to and at the site of intradermal (ID) delivery of H1N1 subunit influenza vaccine (A/California/07/09) (Fig. 1A). We selected PD168393 at a concentration of 4 mM since these conditions have been shown to be immunologically active in murine models and because topical application of other kinase inhibitors are tested in the millimolar range despite having *in vitro* inhibitory concentration 50 (IC50) values in the nanomolar range^14^,^15^,^18^. Serum collected from mice immunized after topical application of an EGFRI (or vehicle control) was assessed for hemagglutination inhibition (HAI) and neutralizing antibody (NT) titers, both of which are considered as correlates of influenza-vaccine induced protective immunity^19^. As a positive control, we included mice that received only the vaccine and no topical treatments. As negative controls we included one group of mice that only received PBS intradermally and one group of mice topically treated with the EGFRI alone (no vaccine). We found that a single application of the EGFRI PD168393 resulted in at least 2-fold higher HAI titers compared to vaccine alone (p = 0.006) and vehicle/vaccine control (p = 0.01) mice as early as week 4 following vaccination (Fig. 1C). These differences were still present at week 8 with p values of 0.04 and 0.064 (using an unpaired two-tailed t-test) when comparing the vaccine/EGFRI group with the vaccine alone and vehicle/vaccine groups respectively (Fig. 1D). Although there were no differences in NT titers between the groups at weeks 2 and 4 post-vaccination (Fig. 1E–F), the NT titers in the EGFRI/vaccine group were 2-fold higher than the vaccine alone control (p=0.04) and 4-fold higher than the vehicle/vaccine group (p = 0.02) by week 8 (Fig. 1E–G). Neither the PBS nor the EGFRI alone negative control groups showed any detectable HAI or neutralizing antibody titers. These data illustrate that in this experimental system, a single topical application of an EGFRI can enhance the generation of functionally relevant antibodies following vaccination.

### EGFRI treatment induces increased numbers of vaccine-specific antibody secreting plasma cells in local lymph nodes

**Figure 2:**
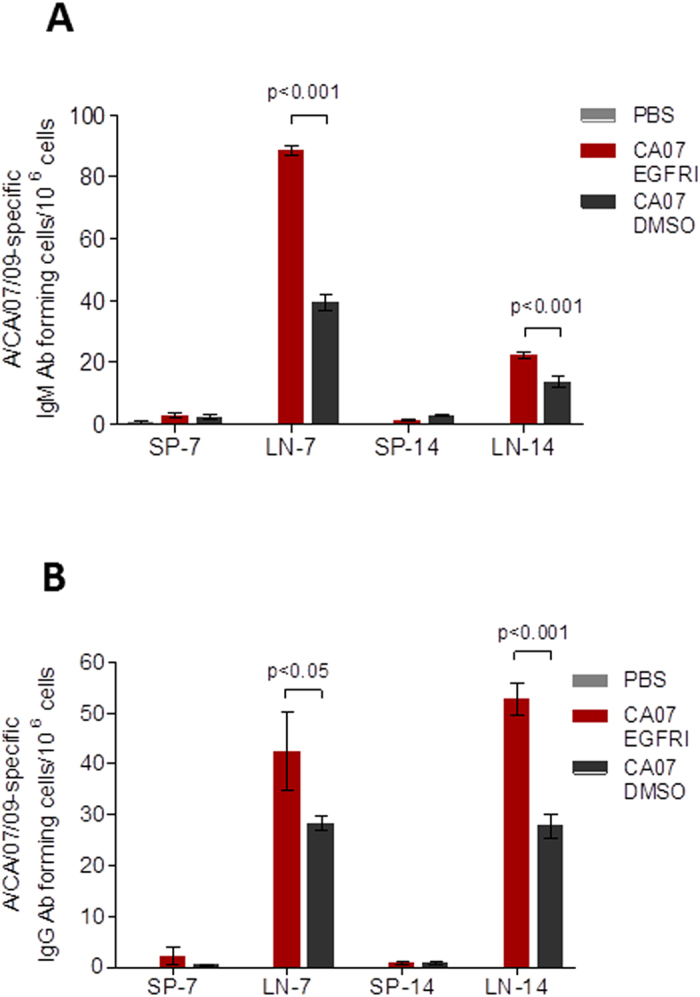
Humoral immune reposes in local lymph nodes and spleens in mice topically pretreated with PD168393 prior to ID vaccination. Cutaneous lymph nodes (LN) and spleens (SP) were collected on day 7 and 14 after immunization. (**A**) Influenza specific IgM and (**B**) IgG secreting cells were quantified by ELISPOT. Values are expressed as mean +/− SEM (n = 4).

In order to determine how the application of PD168393 impacts cellular immune responses following vaccination, we measured influenza-specific IgM^+^ and IgG^+^-secreting plasma cells in the skin draining lymph nodes and the spleen at one and two weeks post-vaccination. The antigen-specific IgM^+^ secreting plasma cells peaked one week after vaccination in both groups although at both time points, the EGFRI/vaccine group exhibited 2-fold higher plasma cell numbers than the vehicle/vaccine group (p < 0.001) (Fig. 2A). The influenza-specific IgG^+^-secreting plasma cells in the EGFRI/vaccine group peaked 2 weeks after vaccination reaching two-fold higher numbers than the vaccine/vehicle cohort (p < 0.001) (Fig. 2B)^20^. No changes in IgG^+^ plasma cell numbers were observed in the vaccine/vehicle cohort during this period. In contrast to the pronounced differences in the local immune responses, the antigen-specific secreting plasma cells were less evident in the spleen and did not show any notable differences between the EGFRI/vaccine and vehicle/vaccine groups, suggesting that the topical treatments are acting locally rather than systemically.

### Topical application of an EGFRI increases cytokine production in skin draining lymph nodes of vaccinated mice.

**Figure 3:**
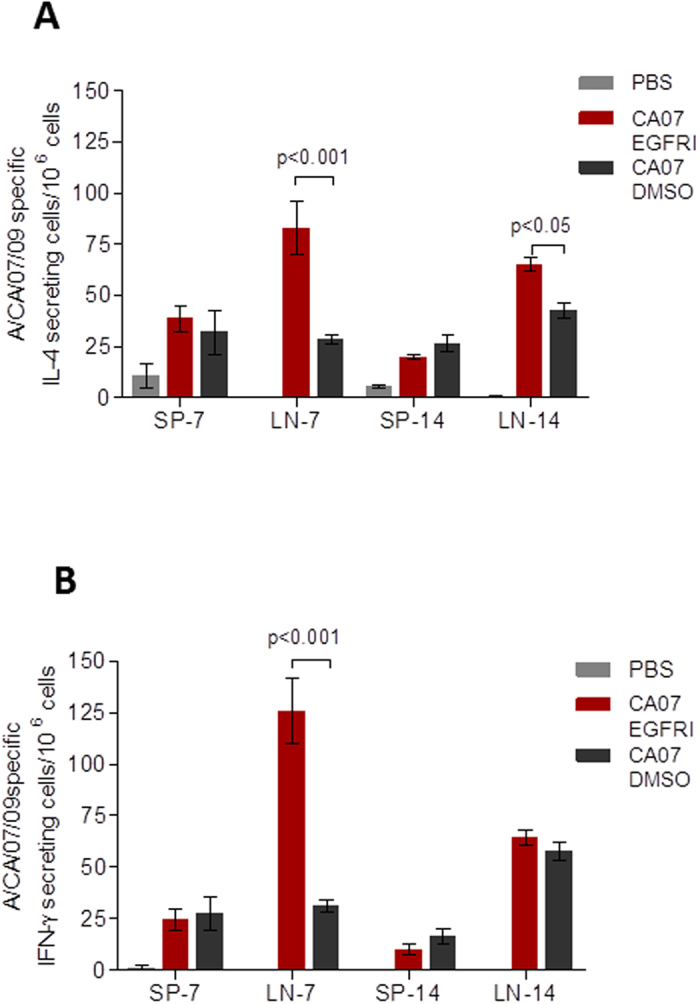
Quantification of IL-4 and IFN-γ secreting cells in local lymph nodes and spleens in mice topically pretreated with PD168393 prior to ID vaccination. Cutaneous lymph nodes (LN) and spleens (SP) were collected on day 7 and 14 after immunization. (**A**) Influenza specific IL-4 and (**B**) IFN-γ secreting cells were quantified by ELISPOT. Values are expressed as mean +/− SEM (n = 4).

To assess the impact of the topically applied EGFRI on cytokine production and immune cell activation, we measured levels of IFN-γ and IL-4 producing influenza-specific lymphocytes in the skin-draining lymph nodes and spleens of vaccinated mice. Both IL-4 and IFN-γ producing influenza-specific cell numbers peaked as early as 7 days in the EGFRI/vaccine group reaching 3-fold and 5-fold differences respectively when compared to the vehicle/vaccine control numbers (p < 0.001) (Fig. 3A,B). The vehicle/vaccine group showed a delayed increase of IL-4 and IFN-γ producing influenza-specific cells although the levels of IL-4 secreting lymphocytes remained significantly lower than the EGFRI/vaccine group throughout the period of 14 days (p < 0.05). Again, in spleens the numbers of these cells were low and no differences were observed between EGFRI- and vehicle-treated mice.

### Skin treatment with an EGFRI prior to vaccination increases protective immunity

**Figure 4:**
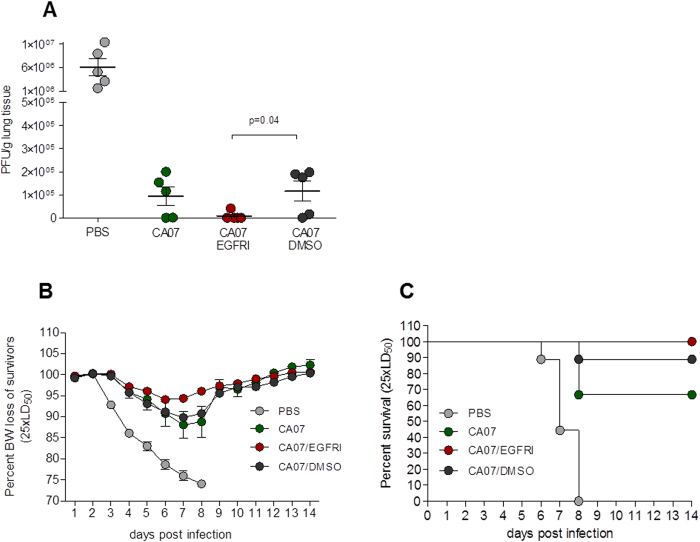
Protective immunity in mice treated with EGFRI prior to vaccination. Immunized groups and control mice were challenged with 25xLD_50_ of mouse adapted A/California 07/09 (H1N1) virus 9 weeks post-immunization. (**A**) Titers of virus in lungs collected 4 days after challenge with 25 x LD_50_ homologous virus was determined by plaque assay (5 mice/group) (**B**) Body weight changes and (**C**) survival rates were monitored for 14 days (n = 9). Values for lung virus titers and body weights are expressed as mean +/− SEM.

To determine the impact of the EGFRI on vaccine-mediated protection against virus challenge, mice were infected with 25xLD_50_ of homologous influenza virus at nine weeks following vaccination. Analysis of pulmonary viral loads revealed that the EGFRI/vaccine group had 12-fold and 14-fold lower titers than the vaccine alone group (p = 0.071) and vehicle/vaccine group respectively (p < 0.04) (Fig. 4A). While the differences between the vaccine alone and the EGFRI/vaccine groups approached but did not reach statistical significance, the EGFRI/vaccine group had the least amount of pulmonary virus detected 4 days post infection.

In a separate cohort of vaccinated mice challenged with the same lethal dose, we observed that while all mice suffered weight losses with a maximum on day 7 post-challenge, the EGFRI/vaccine group exhibited the lowest losses among all groups (7%) and the vehicle/vaccine group suffered the highest losses (12%) (Fig. 4B). Approximately sixty seven percent of mice that received influenza vaccine alone survived infection whereas the EGFRI/vaccine and vehicle/vaccine groups showed 100% and 88.9% survival rates respectively (Fig. 4C). Only the EGFRI/vaccine group differed significantly from the vaccine alone group (p<0.05) supporting the idea that the topically applied EGFRI enhanced protective immunity. These results demonstrate that topical application of a protein kinase inhibitor such as an EGFRI at the site of vaccination can modulate the protective effects elicited by cutaneous vaccination.

### Topical PD168393 application alters CCL2 RNA expression within the skin.

**Figure 5:**
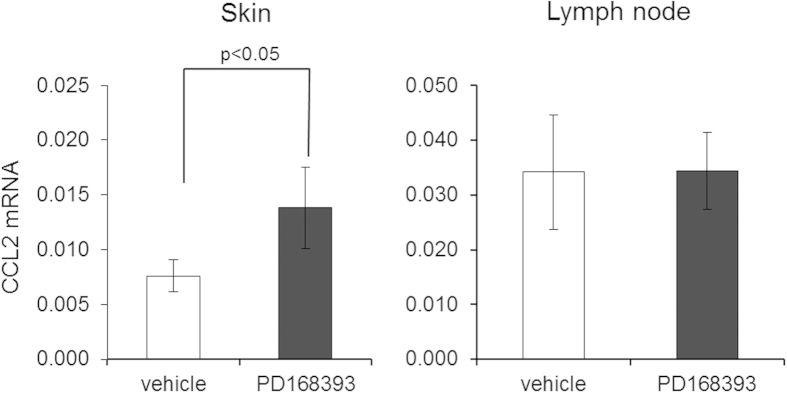
Steady state mRNA levels of CCL2 are increased within the skin 6 hours following topical application of PD168393. Real-time RT-PCR was used to quantify steady state mRNA levels of CCL2 within RNA from skin (left panel) or inguinal lymph nodes (right panel) six hours following the application of the irreversible EGFR inhibitor PD168393 (or vehicle control). Five mice per group were used. The *y*-axis represents the mean normalized expression (relative to GAPDH) and error bars represent the standard deviation.

To determine how EGFR inhibition alters the expression of genes that are relevant to cutaneous immune responses in our model, we applied PD168393 or vehicle to the dorsum of BALB/c mice (5 mice per group) and measured steady state mRNA levels of several genes including those previously shown to be altered by EGFRI therapy. We selected a six-hour time point for these experiments because prior studies have shown that cellular responses to vaccination occur within hours and that PD168393 enhances the expression of the aforementioned chemokines at similar time points^15^. Changes in RNA expression were only considered meaningful if the differences were maintained with each of the three housekeeping genes used in the analysis. As shown in Fig. 5, using this criteria, we found that the topical application of PD168393 to the dorsal skin of BALB/c mice leads to a statistically significant increase in steady state mRNA levels of the chemokine CCL2 (also known as monocyte chemoattractant protein (MCP-1) within the skin. In contrast, steady state levels of the housekeeping genes and several other genes analyzed were unchanged by topical PD168393 at this time point in the skin and the skin draining lymph nodes (Supplementary Tables 1 and 2). These studies demonstrate that a single application of an EGFRI to mouse skin can alter the expression of a chemoattractant protein^21^. In addition to these changes in gene expression, we observed an increase in epidermal thickness 24 and 48 hours after PD168393 application as well as an increase in MHC class II positive cells within the dermis consistent with the ability of the topically applied EGFRI to disrupt the epidermal differentiation program and alter immune cell trafficking into the skin (Supplementary Figures 1 and 2).

## Discussion

In this study, we demonstrate that topical application of an EGFR kinase inhibitor can enhance the humoral and cellular response to influenza vaccination and augment protection against influenza challenge. These findings have several important implications. Firstly, to our knowledge, they provide the first proof-of-concept evidence that the response to vaccination can be modulated using a topically applied kinase inhibitor. In addition, our data also suggest that cellular events within regional lymph nodes that drain the skin can be modulated using this approach. While our study demonstrates the relevance of this approach in the context of an influenza vaccination, it may be more broadly applicable and relevant because at present there are few options available to pharmacologically modulate events within the lymph node basins draining epithelial tissues; sites that are critical during the early stages of antigen presentation and T cell activation against infections and malignancies.

We selected an inhibitor of the EGFR pathway for these initial studies because prior studies have demonstrated that EGFRIs can enhance the elicitation phase of contact hypersensitivity and increase the expression of several chemokines such as CCL-2 (MCP-1), CXCL-10 (IP-10) and CCL27^15^,^22^. Interestingly, some of these chemokines were also shown to be induced by intradermal immunization^23^. In addition, EGFRIs have also been shown to augment immune cell trafficking into the skin, increase the expression of genes involved in antigen presentation and inhibit the function of key cellular elements of the immune system namely regulatory T cells^13^,^24^,^25^. Thus, pre-clinical and clinical studies of EGFRIs have illustrated that these drugs have effects on cutaneous immune homeostasis and other factors that likely influence the response to vaccination.

Many of the aforementioned effects of EGFRIs likely contribute to the unwanted skin rash that occurs in patients during systemic therapy for cancer^26^. However, when topically applied, some of these effects may be valuable and exploitable to enhance cutaneous immune responses. The data presented herein provide evidence for this concept by using a topically applied EGFRI in the context of vaccination; in effect, as an adjuvant. The use of such inhibitor-based adjuvants alone or in combination with other adjuvants may represent a novel approach to modulate the response to vaccination. By inhibiting defined signaling pathways that govern the expression of genes regulating cutaneous immune homeostasis, the quantitative and/or qualitative nature of the cellular response to deposited vaccine components may be modified. Repurposing small molecule kinase inhibitors in this manner provides several potential opportunities that are not possible with more conventional adjuvants as discussed in more detail below.

At the most basic level, the enormous efforts that have gone into the development of targeted therapies for the treatment of cancer have resulted in an expanding number of kinase inhibitors that are FDA approved for systemic use in humans. Thus, there is pre-existing information regarding the safety of these compounds at dosage levels that are much higher than would be needed for locally delivered applications. Likewise, in contrast to more conventional adjuvants where mechanistic details are largely lacking, there is detailed mechanistic information available for kinase inhibitors in terms of their molecular targets. Another potential advantage of kinase inhibitors stems from the fact that the size of these compounds is small relative to that of more conventional adjuvants or immunostimulatory molecules such as chemokines. As such, these molecules will likely be amenable to newer vaccination approaches such as aerosolized delivery systems and epicutaneous vaccination approaches. While our studies focused on an EGFR inhibitor, it is possible that targeting other kinases within the skin may have similar or even more robust effects on vaccination responses.

Our study has several limitations that deserve mention. For example, while PD168393 is highly active against the EGFR, it is also active against other kinases such as ErbB-2^27^. Therefore, we cannot exclude the possibility that inhibition of other kinases may be contributing to the effects we observed. In addition, the application of DMSO (in ethanol) is unlikely to be immunologically null. Indeed, DMSO has been reported to enhance the penetration of compounds into the skin, and impact cutaneous immune cell recruitment, wound healing and tumorigenesis^28^,^29^,^30^,^31^,^32^. Thus, there may be some impact of the vehicle used in our experimental model. Likewise, while we observed no alterations in RNA levels within the skin-draining lymph nodes when PD168393 was topically applied (in contrast to the increase in CCL2 mRNA in the skin), we cannot entirely exclude the possibility that the topical application of the EGFRI had some systemic effect that was not evaluated in our analysis. Another important point worthy of mention is that at present EGFR inhibitors are only approved for use systemically not topically. Thus, while our data provide proof-of-concept evidence that topical application of an EGFRI can influence vaccine responses within murine skin, additional studies are needed to see the effects of topical EGFRIs on human skin.

Despite the aforementioned limitations, to our knowledge this is the first report in which a small molecule kinase inhibitor was re-purposed in this manner; namely as a vaccine adjuvant. While there is increasing recognition of the immune effects of targeted therapies such as EGFRIs, our data provide proof-of-concept evidence illustrating that some of these immune effects can be exploited locally to enhance the induction of antigen-specific immune responses. While the present study centers on the immune response to vaccination, this same approach may hold value to enhance local immune responses to infections and cancers of the skin and other tissues such as those of the mucous membranes that are accessible to locally delivered therapies.

## Materials And Methods

### Cells and virus stocks

Madin-Darby canine kidney (MDCK) cells (CCL 34, ATCC, Manassas, VA) were maintained in Dulbecco’s Modified Eagle’s Medium (Mediatech, Herndon, VA) containing 10% fetal bovine serum (Hyclone, Thermo Scientific, Rockford, IL). Influenza virus stocks (H1N1 A/California/07/09) were propagated in MDCK cells and purified by sucrose gradient centrifugation. The purity of the virus was determined by SDS PAGE followed by Coomassie blue staining^12^. Inactivation of purified virus with 0.1% formalin (v/v) was confirmed by plaque assay in MDCK cells. The hemagglutination (HA) activity was determined using turkey blood cells (LAMPIRE, Pipersville, PA)^33^. Since A/California/07/09 virus was used for infection of vaccinated and unvaccinated BALB/c mice we determined the lethal dose fifty (LD_50_) with the Reed-Munch formula^34^.

### Immunizations, challenge and sample collection

Six- to eight-week-old female BALB/c mice were purchased from Harlan Laboratories (Tampa, FL) and housed at Emory University Whitehead Animal Facility. Dorsal hair for intradermal vaccination was removed under systemic anesthesia with xylazine/ketamine cocktail. On the day of immunization 40 μl of 4 mM PD168393 (VWR International, Dallas, TX) or vehicle (DMSO and ethanol/negative control) were applied topically on shaved skin and allowed to dry for 10 min prior to intradermal injection of subunit influenza vaccine (H1N1 A/California/07/09), 2 μg total protein in 50 μl PBS or PBS only (negative control administered intradermally) in the same area. Some mice received only a topical treatment and were not vaccinated as indicated in the text. Animals were bled (via submandibular puncture) at weeks 2, 4 and 8 after immunization and spleens, inguinal and axillary lymph nodes were collected at weeks 1 and 2 from euthanized mice for evaluation of cellular immune responses^35^. Lungs were collected on day 4 post-infection and lung homogenates were stored in DMEM with 1% Penicillin/Streptomycin at −20 °C until assayed for viral titers. Inguinal and axillary lymph nodes and spleens were processed similarly into single cell suspensions in complete RPMI 1640 for cytokine determination. Spleens were treated with red blood cell lysis buffer after their initial processing (Sigma, St. Louis, MO). For challenge, mice were infected intranasally under isoflurane anesthesia with 25xLD_50_ (47 PFU) mouse adapted virus 9 weeks after vaccination and monitored for 14 days for body weight changes, fever, hunched posture, and mortality. Weight loss exceeding 25% was used as the experimental end point, at which mice were euthanized according to IACUC guidelines. All studies were approved by Emory University’s Institutional Animal Care and Use Committee.

### Humoral immune responses

Influenza-specific hemagglutination inhibition titers (HAI) were assessed according to the WHO protocol^36^. The HAI titer was read as the reciprocal of the highest dilution of serum that conferred inhibition of hemagglutination. Neutralizing antibody titers were determined in heat inactivated sera by microneutralization assay using 100 TCID_50_/well of A/California/07/2009 virus^37^. The NT was read as the reciprocal of the highest dilution that inhibited MDCK cell infection by the virus. The values were expressed as the geometric mean+/−standard error of the mean.

### Cellular immune responses

Freshly isolated splenocytes and lymphocytes (1.0 × 10^6^/200 μl cRPMI) were cultured for 36-48 h in the presence of 4 μg/ml A/California/07/09 influenza vaccine to enumerate vaccine-specific IL-4 and IFN-γ ELISPOT secreting cells. ELISPOT reagents were purchased from BD-PharMingen (San Jose, CA). Enumeration of vaccine-specific cytokine secreting cells was carried out by ELISPOT assay and counted using an ELISPOT reader and counter (Cellular Technologies, Shaker Heights, OH).

### Post-challenge lung titers

Lung homogenates were prepared in DMEM and viral titers were assessed per gram of tissue by plaque assay^38^.

### Gene expression assessment by real-time RT-PCR

Six hours following the application of 40 μl of vehicle (10% DMSO in ethanol) or PD168393 (4 mM) to the backs of mice, mice were euthanized and the treated areas of skin excised and placed in RNAlater (Qiagen, Valencia, CA). Tissue samples were processed with the Qiagen miRNEAsy mini Kit 183 (Qiagen) and TissueLyser II bead mill (Qiagen,) according to the manufacturers’ protocols. Total RNA was quantitated on a ThermoScientfic NanoDrop 1000 and integrity assessed on an Agilent 2100 Bioanalyzer using the Agilent RNA 6000 Nano Kit (Agilent, Santa Clara, CA). 10 μg of total RNA was converted to cDNA using the Applied Biosystems (Grand Island, NY) High-Capacity cDNA Archive Kit according to the manufacturer’s protocol.

Quantitative real-time PCR was performed on a Bio-Rad CFX96 thermocycler (Bio-Rad, Herdules, California) and normalized expression levels calculated using the ΔΔCt mode of CFX Manager Software (Bio-Rad, Hercules, CA). Levels of steady state mRNA of the following genes was assessed: chemokine (C-C motif) ligand (CCL) 2, CCL5, MHC class II transactivator (CIITA), chemokine (C-X-C motif) ligand (CXCL) 10, epidermal growth factor receptor (EGFR), murine MHC class I and II genes (H2Dd, H2IA, H2IE, H2K), interferon regulatory factor (IRF) 1, NLR family CARD domain containing (NLRC) 5. Steady state mRNA levels of glyceraldehyde-3-phosphate dehydrogenase (GAPDH), hypoxanthine phosphoribosyltransferase (HRPT) 1 and peptidylprolyl isomerase A (PPIA) were also measured. All primers were purchased from *Real Time Primers* LLC, Elkins Park, *PA* and sequences are available upon request.

### Histopathology

Twenty four and 48 hours following application of PD168393 or vehicle, mice were euthanized and dorsum skin tissue samples excised and fixed in buffered formalin solution, embedded in paraffin, sectioned, and the slides stained with hematoxylin and eosin. The stained slides were scanned and reviewed digitally as well as using light microscopy.

### Immunofluorescence

Mouse ears were collected 24 and 48 hours after topical application of EGFRI or vehicle in Tissue-Tek® O.C.T. Compound, (Sakura® Finete, VWR) and stored at −80 ^o^C until further use. Five micron skin cryosections were fixed in cold acetone and incubated with PerCP anti-mouse I-A/I-E clone M5/114.15.2 (Biolegend, San Diego, CA) diluted in PBS with 2% bovine serum albumin, followed by VECTASHIELD mounting medium with DAPI (Vector Laboratories Inc. Burlingame, CA) to stain the nuclei. The stained samples were examined under fluorescence microscopy.

### Statistics

For humoral immune responses (HAI and NT) a two-tailed unpaired Student’s *t*-test and a one-way ANOVA including Bonferroni’s multiple comparison test were utilized. Two-way ANOVA test was used for the cell-based immune responses. For lung titers and body weight changes we applied two-tailed unpaired Student’s *t*-test. For the survival curves, statistics were calculated using a Log-rank (Mantel-Cox) test. A p-value less than 0.05 was considered significant. Means and standard deviations were calculated from quadruplicate runs and at least two independent animal experiments. The statistical significance of differences for real-time PCR data was calculated using a two-tailed unpaired Student’s *t*-test for normally distributed data and a Mann-Whitney test when data was not normally distributed as determined by the method of Komolgorov and Smirnov.

## Additional Information

**How to cite this article**: Pulit-Penaloza, J. A. *et al*. Modulation of influenza vaccine immune response using an epidermal growth factor receptor kinase inhibitor. *Sci. Rep*. **5**, 12321; doi: 10.1038/srep12321 (2015).

## Supplementary Material

Supplementary Information
