# Supplementary material for: Modulation of influenza vaccine immune responses using an epidermal growth factor receptor kinase inhibitor
Source: Sci Rep. 2015 Jul 31;5:12321. doi: 10.1038/srep12321 (PMC4521188; doi:10.1038/srep12321)
Supplement: Supplementary Information [file srep12321-s1.pdf]

Supplementary Information for

‘Modulation of influenza vaccine immune responses using an epidermal growth factor receptor kinase inhibitor’

Joanna A. Pulit-Penaloza, Bishu Sapkota, E. Stein Esser, Richard W. Compans,

Brian P. Pollack and Ioanna Skountzou

Supplementary Table 1. The impact of PD168393 on gene expression in the skin[a].

Gene symbol	vehicle	PD168393	*P* value
Mean expression relative to GAPDH			
Ccl2	0.007608	0.01383	0.0084 (*t*-test)
Ccl5	6.2 x10-5	5.6x10-5	0.9153 (M-W test)
Cxcl1910	0.03066	0.04340	0.0588 (*t*-test)
H-2K[d]	0.1145	0.1195	0.7752 (*t*-test)
Irf1	0.04684	0.07177	0.0105 (*t*-test)
Ciita	0.00395	0.004594	0.1607 (*t*-test)
Egfr	0.002256	0.002324	0.8397 (*t*-test)
Nlrc5	0.01773	0.01949	0.4041 (*t*-test)
B2m	0.4122	0.4231	0.8159 (*t*-test)
H2Dd	0.06282	0.08427	0.1276 (*t*-test)
Ifng	2.2x10-5	3.4x10-5	0.3706 (*t*-test)
I-A	0.003184	0.003848	0.1339 (*t*-test)
I-E	0.001978	0.002642	0.0952 (M-W test)
Ppia	0.08808	0.09280	0.5476 (*t*-test)
Hprt	0.006626	0.007206	0.6905 (*t*-test)
Mean expression relative to PPIA			
CCL2	0.1034	0.2077	0.0367 (*t*-test)
Irf1	0.6133	0.8523	0.1328 (*t*-test)
Gapdh	11.845	11.073	0.6040 (*t*-test)
Hprt	0.07735	0.07979	0.8244 (*t*-test)
Mean expression relative to HPRT			
Ccl2	1.332	2.549	0.0143 (*t*-test)
Irf1	29.195	41.477	0.0725 (*t*-test)
Gapdh	156.48	138.79	0.3078 (*t*-test)
Ppia	13.501	12.879	0.7247 (*t*-test)

[a] Real-time RT-PCR was used to measure the expression of the indicated genes six hours following the application of vehicle or PD168393 as indicated. Values represent the mean normalized expression from each group (five mice per group) relative to the housekeeping gene indicated. *P* values were calculated using the Student's *t*-test (t-test) for normally distributed data or Mann-Whitney test (M-W test) for data that failed normality testing.

Supplementary Table 2. The impact of PD168393 on gene expression within lymph nodes[a]

Gene symbol	vehicle	PD168393	*P* value
Mean expression relative to GAPDH			
Ccl2	0.0342	0.0344	0.9753 (*t*-test)
Ccl5	0.0523	0.0588	0.3928 (*t*-test)
Cxcl1910	0.1389	0.1751	0.1384 (*t*-test)
H-2K^d	0.9228	0.7586	0.1759 (*t*-test)
Irf1	0.5217	0.4024	0.0237 (*t*-test)
Ciita	0.1467	0.1172	0.0361 (*t*-test)
Egfr	0.0006	0.0006	0.8122 (*t*-test)
Nlrc5	0.1339	0.1062	0.0144 (*t*-test)
B2m	0.4122	0.4231	0.8159 (*t*-test)
H2Dd	0.4502	0.4479	0.9742 (*t-test*)
Ifng	0.0007	0.0006	0.5443 (*t*-test)
I-A	0.0733	0.0686	0.4302 (*t*-test)
I-E	0.0560	0.0518	0.8653 (*t*-test)
Ppia	0.2694	0.2213	0.1242 (*t*-test)
Hprt	0.0039	0.0031	0.0710 (*t*-test)
Mean expression relative to PPIA			
CIITA	0.0017	0.0018	0.4945 (*t*-test)
Irf1	2.6520	2.5940	0.6905 (M-W test)
Gapdh	3.7890	4.7290	0.1760 (*t*-test)
Hprt	0.0147	0.0141	0.3339 (*t*-test)
NLRC5	1.4940	1.5650	0.4522 (*t*-test)
Mean expression relative to HPRT			
Irf1	181.20	183.80	0.8359 (*t*-test)
Gapdh	257.13	336.32	0.0952 (M-W test)
Ppia	68.367	70.847	0.3458 (*t*-test)

[a] Real-time RT-PCR was used to measure the expression of the indicated genes six hours following the application of vehicle or PD168393 as indicated. Values represent the mean normalized expression from each group (five mice per group) relative to the housekeeping gene indicated. *P* values were calculated using the Student's *t-test* (*t-test*) for normally distributed data or Mann-Whitney test (M-W test) for data that failed normality testing.

Supplementary Figure 1

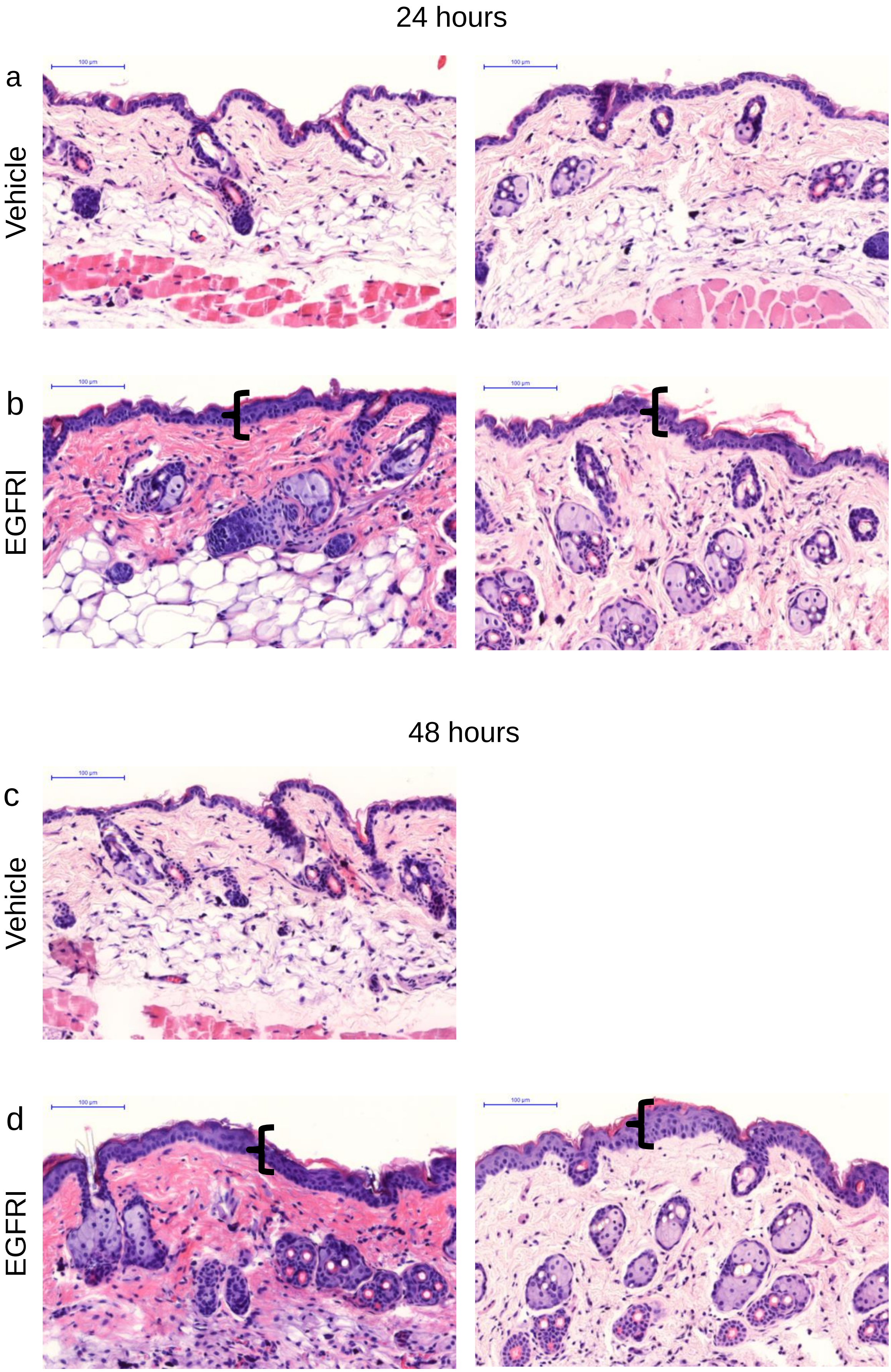

Supplementary Figure 2

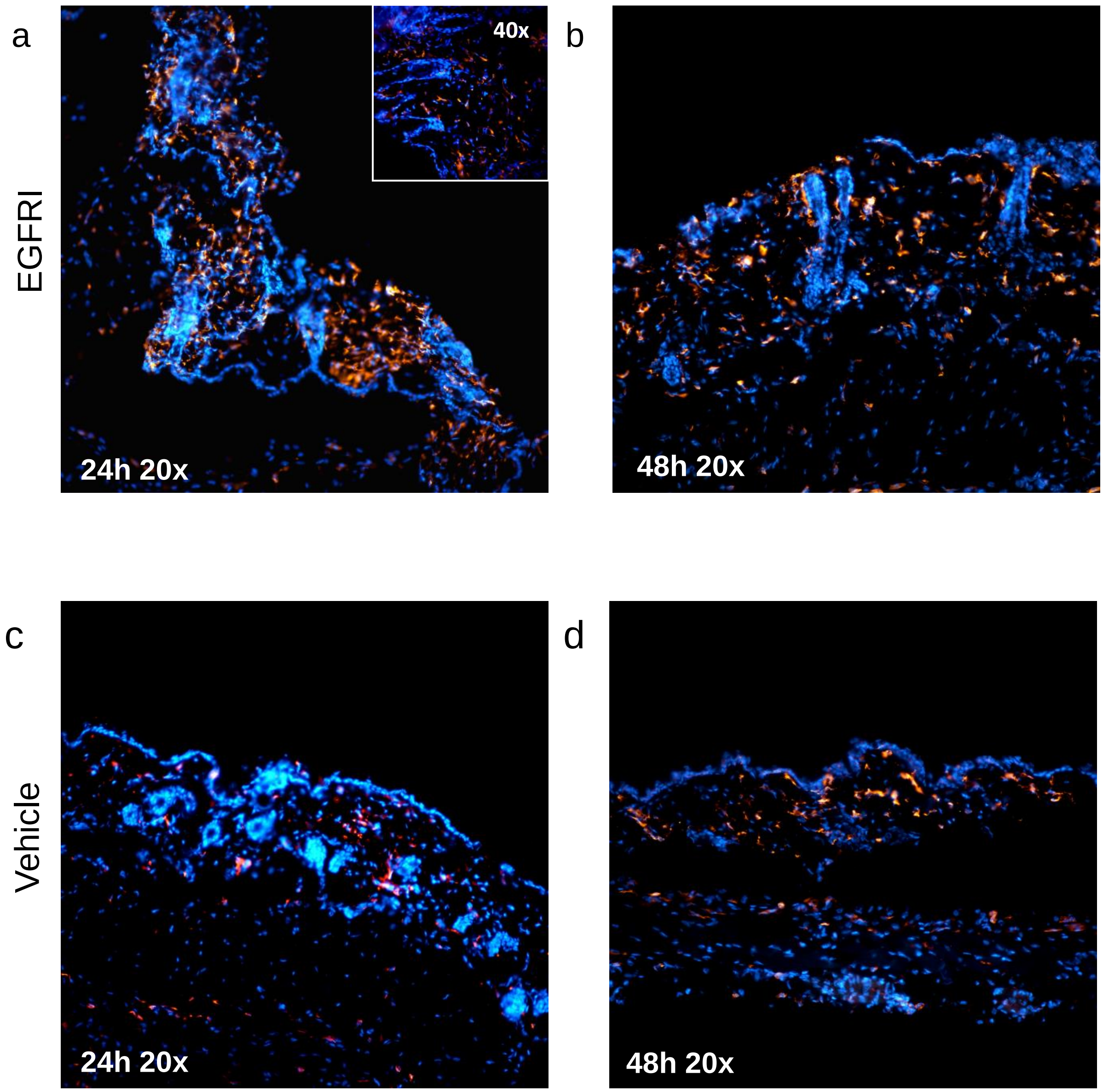

Supplementary Figure 1. Hematoxylin and eosin-stained slides of formalin fixed, paraffin embedded skin 24 or 48 hours after topical application of EGFRI or vehicle. Each panel depicts one mouse. Magnification 20x. Black left braces are placed next to the thickened epidermis in panels from PD168393-treated skin.

Supplementary Figure 2. Immunofluorescent staining of PD168393- or vehicle-treated skin using a PerCP-conjugated anti-mouse I-A/I-E antibody with DAPI counterstain. Mouse ears were topically treated with either PD168393 or vehicle and were collected 24 and 48 h later following euthanasia. (A) PD168393 at 24 h and (B) 48 h; (C) vehicle at 24 h and (D) 48 h. Each panel depicts one mouse. Magnification 20x. The insert image in panel (A) shows the skin section at 40x magnification.
